# *In vitro* anti-gastrointestinal cancer activity of *Toxocara canis* *-*derived peptide: Analyzing the expression level of factors related to cell proliferation and tumor growth

**DOI:** 10.3389/fphar.2022.878724

**Published:** 2022-09-20

**Authors:** Saeed Bahadory, Javid Sadraei, Mohammad Zibaei, Majid Pirestani, Abdolhossein Dalimi

**Affiliations:** ^1^ Department of Parasitology, Faculty of Medical Sciences, Tarbiat Modares University, Tehran, Iran; ^2^ Department of Parasitology and Mycology, School of Medicine, Alborz University of Medical Sciences, Karaj, Iran; ^3^ Evidence-Based Phytotherapy and Complementary Medicine Research Center, Alborz University of Medical Sciences, Karaj, Iran

**Keywords:** anti-cancer, *Toxocara canis*, peptide, gastrointestinal, real-time PCR

## Abstract

**Background:** Recently, a hypothesis about the negative relationship between cancers and parasites has been proposed and investigated; some parasitic worms and their products can affect the cancer cell proliferation. Due to the potential anti-cancer effect of helminthic parasites, in the present study, the excretory–secretory protein of *Toxocara canis* (*T. canis*) parasite was used to evaluate the possible anti-cancer properties and their effect on gastrointestinal and liver cancer cell proliferation-related genes in laboratory conditions.

**Methods and materials:** The selected synthesized peptide fraction from the *T. canis* excretory–secretory Troponin protein peptide (ES TPP) was exposed at 32, 64, 128, and 256 μg/ml concentrations to three gastrointestinal cancer cell lines AGS, HT-29, and Caco 2, as well as HDF cells as normal cell lines. We used the MTT assay to evaluate cellular changes and cell viability (CV). Variations in gene (Bcl-2, APAF1, ZEB1, VEGF, cyclin-D1, and caspase-3) expression were analyzed by real-time RT-PCR.

**Results:** After 24 h of exposure to pept1ides and cell lines, a decrease in CV was observed at a concentration of 64 μg/ml and compared to the control group. Then, after 48 h, a significant decrease in the CV of Caco 2 cells was observed at a concentration of 32 μg/ml; in the other cancer cell lines, concentrations above 32 μg/ml were effective. The peptide was able to significantly alter the expression of the studied genes at a concentration of 100 μg/ml.

**Conclusion:** Although the studied peptide at high concentrations could have a statistically significant effect on cancer cells, it is still far from the standard drug and can be optimized and promising in future studies.

## 1 Introduction

Cancers are a worldwide life-threatening concern, which still imposes heavy health and economic impairment every year (Jones and Baylin, 2007; Khatami et al., 2022). Approximately 20 million new cancerous cases and nearly 10 million deaths are reported annually (Sung et al., 2021). Among the cancer causes, tobacco use, high body mass index, genetic background, stress, and some infections are the chief risk factors that have been defined (Wu et al., 2016; Nahand et al., 2021). Amid infectious agents, a few viruses, such as BLV, papillomavirus, and hepatitis, also certain parasites such as *Schistosoma haematobium*, have been linked to cancers (Khatami et al., 2020; Khatami et al., 2021; Khatami et al., 2022).

In recent years, owing to the immunomodulatory property of parasites (mostly helminthes), a negative relationship between cancer and parasitic infection has been investigated and reported (Darani and Yousefi, 2012; Callejas et al., 2018). It has been shown that some chronically parasitic infections and/or their excretory–secretory compounds can regulate and manipulate the host immune system (McSorley et al., 2013; Dos-Santos et al., 2016; Zibaei et al., 2019b).

*Toxocara canis* is a dog-specific nematode that can also infect humans zoonotically (Ma et al., 2018; Zibaei et al., 2019a). The third-stage larvae of this roundworm in humans are infectious agents; humans are infected by infective egg ingestion with vegetables or pica, as well as contaminated paratenic host consumption (Despommier, 2003; Rasouli et al., 2020). Although toxocariasis often presents in covert form and is asymptomatic, symptomatic cases often manifested as visceral larva migrans (VLM) and ocular larva migrans syndrome (Rubinsky-Elefant et al., 2010). *T. canis* larva-secreted compounds have been shown to contain a wide range of proteins that have the potential to impact on the immune system (Yamasaki et al., 2000; da Silva et al., 2018). On the other hand, due to more establishments of parasitic larvae in the intestine and liver, the effects of parasitic secretions on these tissue cells probably are traceable (Morales-Yanez et al., 2019).

Up to now, some parasitic products have been shown to reduce or inhibit proliferation, as well as induce apoptosis in cancer cells (Darani et al., 2009; Chookami et al., 2016). By evaluating cell viability and metastatic/angiogenesis and apoptosis-related factors such as *Bcl-2* (B-cell lymphoma 2), *APAF1* (apoptotic protease activating factor-1), ZEB1 (zinc finger E-box binding homeobox 1), VEGF (vascular endothelial growth factor), cyclin-D1, and caspase-3 in cells, the effect of parasitic products can be measured (Forma and Bryś, 2021).

In this regard, the proteome composition of *T. canis* excretory–secretory antigens has been identified, and according to the findings of computer-based analyses at the (http://crdd.osdd.net/raghava/cancerppd/index.php) site, part of the *T. canis*-secreted protein with the 18 amino acids showed a high degree of similarity (≈93%) to other anti-cancer agents (unpublished data).

In the present study, we investigated the anti-cancer potential of *T. canis* compounds in different gastrointestinal and liver cancer cell lines.

## 2 Materials and methods

### 2.1 Ethical approval

All experimental procedures of the current study were reviewed and approved by the ethics committee of Tarbiat Modares University (IR.MODARES.REC.1400.111).

### 2.2 Peptide synthesis

Among the identified proteomes of *T. canis* excretory–secretory antigens, part of TOXCA Troponin T protein with the RNRQEEEQRRQRREADRL amino acid sequence was selected. Following the nominated sequence after analysis in the anti-cancer peptide database and confirmation of the anti-cancer potential of the selected sequence, peptide synthesis was performed by Elab Science (United States, lot no: YZIGY9RHUD) with a purity of over 97.5% and 2425.5 M.W. The synthesized peptide was diluted with ultra-pure water, according to the manufacturer’s protocol, and was ready to use in various concentrations for CV and molecular assay.

### 2.3 *In vitro* anticancer assay

#### 2.3.1 Cell line culture and treatments

AGS, HT-29, Caco 2, and human gastric and colon cancer cell lines, as well as HDF (Primary human dermal fibroblasts) cells as control cells, were obtained from the Iranian Biological Resource Center and cultured in Dulbecco’s modified Eagle’s medium (DMEM)/F12 (Bio Idea Co, Iran) complemented with 10% FBS (Gibco: Thermo Fisher Scientific) and 2 mmol/L L-glutamine (Bio Idea Co, Iran). All cell lines were grown in DMEM/F12 which was supplemented with GlutaMAX, NaHCO_3_, and 15 mM HEPES at 37°C in a humidified atmosphere of 5% CO_2_.

#### 2.3.2 Cell viability determination by MTT assay

Initially, after ensuring 90% confluence of cells by microscopic counting (stained with trypan blue), to assess cell viability measurement by MTT [3-(4, 5-dimethylthiazol-2-yl)-2, 5-diphenyltetrazolium bromide] assay, in 96-well culture plates, approximately 20 × 10^3^ cells/well were seeded in each well containing 200 μl of DMEM medium. In order to determine the adherence of cells to the floor of plate wells, the plates were incubated for 24 h at 37°C and 5% carbon dioxide condition. AGS, HT-29, and Caco 2 cells were exposed and incubated with *T. canis* peptides at 32, 64, 128, and 256 µg/ml increasing concentrations for 24 and 48 h in 96-well culture plates. Regorafenib—an oral multi-kinase inhibitor exhibiting anti-angiogenic activity that is used against gastrointestinal and colorectal cancers—was purchased (Baran Chemical and Pharmaceutical Co, Iran) and applied as a control group. Cell growth inhibition was assessed by the spectrophotometric measurement of the mitochondrial dehydrogenase activity using an MTT assay.

After 24 and 48 h of exposure to peptides and cell lines, 20 μl volume of 5 μg/ml MTT solutions were added to the wells, and the plates were incubated at 37°C again for 4 h. The wells were then emptied, and 200 μl of dimethyl sulfoxide (DMSO) was added to all wells; in this method, viable cells reduce the MTT reagent to the formazan crystalline product (purple color) because they contain NAD(P)H-dependent oxidoreductase enzymes. The produced purple color intensity is directly dependent on the number of living cells and their metabolism. As mentioned, untreated and regorafenib-treated cells were used as controls. The optical density (OD) of the wells was measured using a microplate spectrophotometer (BioTek-ELX800, United States) at 570 nm, 24 and 48 h after exposure. All the experiments were performed in triplicate for each concentration and cell line.

### 2.4 Gene expression analysis (Bcl-2, APAF1, ZEB1, VEGF, cyclin-D1, and caspase-3)

The SYBR green-based quantitative real-time RT-PCR technique was applied to protein and gene expression evaluation. The genomic content **(**total RNA) was extracted from all cultured cell lines to measure the mRNA expression of the Bcl-2, APAF1, ZEB1, VEGF, cyclin-D1, and caspase-3 genes using QIAzol RNA (Qiagen, United States), according to the manufacturer’s protocol. Harvested RNAs’ quantity, purity, and optimum concentration were measured using a nanodrop spectrophotometer (Thermo Scientific^™^ NanoDrop 2000c spectrophotometer) in 260/280 nm ratio, and the RNAs were reverse transcribed to cDNA using RT-specific primers and GAPDH, which was used as a reference gene to normalized the data (Table 1). Real-time RT-PCR was performed in a LightCycler^®^ 96 thermal cycler (Roche, Germany). The final volume for each reaction was a 20 µl mixture consisting of 8 µl of SYBR Green I Master mix, 1 µl of cDNA (as template), and 10 pmol of each primer, as well as 8 µl of nuclease-free distilled water. In the amplification program, first denaturation was carried out at 95°C for 8 min, 40–45 cycles with denaturation a 95°C for 10 s, annealing at 58–62°C for 5 s, and extension at 72°C for 20 s.

**TABLE 1:** Sequences of used primers for the amplification reaction.

Gene	Primer sequence (5′… 3′)	Reference
*Bcl-2*	F: TCG​CCC​TGT​GGA​TGA​CTG​A	Pan et al. (2015)
	R: CAG​AGA​CAG​CCA​GGA​GAA​ATC​A	
*APAF1*	F: TTAGGAGCCAGGTGCGGT	Sun et al. (2012)
	R: GCT​TGT​CTT​TCT​TCC​CAT​TTT​TC	
ZEB1	F: GGC​ATA​CAC​CTA​CTC​AAC​TAC​GG	Wang et al. (2018a)
	R: TGG​GCG​GTG​TAG​AAT​CAG​AGT​C	
*VEGF*	F: TGC​AGA​TTA​TGC​GGA​TCA​AAC​C	Wang et al. (2018b)
	R: TGC​ATT​CAC​ATT​TGT​TGT​GCT​GTA​G	
*Cyclin-D1*	F: CTG​AGG​AGC​CCC​AAC​AAC​TT	Wang et al. (2021)
	R: CAG​TCC​GGG​TCA​CAC​TTG​AT	
*Caspase-3*	F: TTC​AGA​GGG​GAT​CGT​TGT​AGA​AGT​C	Winter et al. (2001)
	R: CAA​GCT​TGT​CGG​CAT​ACT​GTT​TCA​G	
*GAPDH*	F: ACG​GAT​TTG​GTC​GTA​TTG​GG	Liu et al. (2013)
	R: TGA​TTT​TGG​AGG​GAT​CTC​GC	

### 2.5 Statistical analysis

In the present study, all experiments were repeated three times. The treated groups were compared and analyzed statistically with each other and the control group. Data were statistically analyzed using the comparative Ct (ΔΔct) method; also, the real-time PCR findings were first pre-processed, and then, Kruskal–Wallis and Mann–Whitney *U* tests were used to evaluate the differences in the expression level of selected genes (*Bcl-2*, *APAF1*, *ZEB1*, *VEGF*, *cyclin*-D1, and *caspase-3*) among study groups, and the *p*-value < 0.05 was considered to be statistically significant. All analyses were completed by GraphPad Prism v 6.0 software.

## 3 Results

### 3.1 *T. canis* excretory–secretory troponin protein peptide effects on mortality and proliferation in cancer and normal cell lines

**FIGURE 1:**
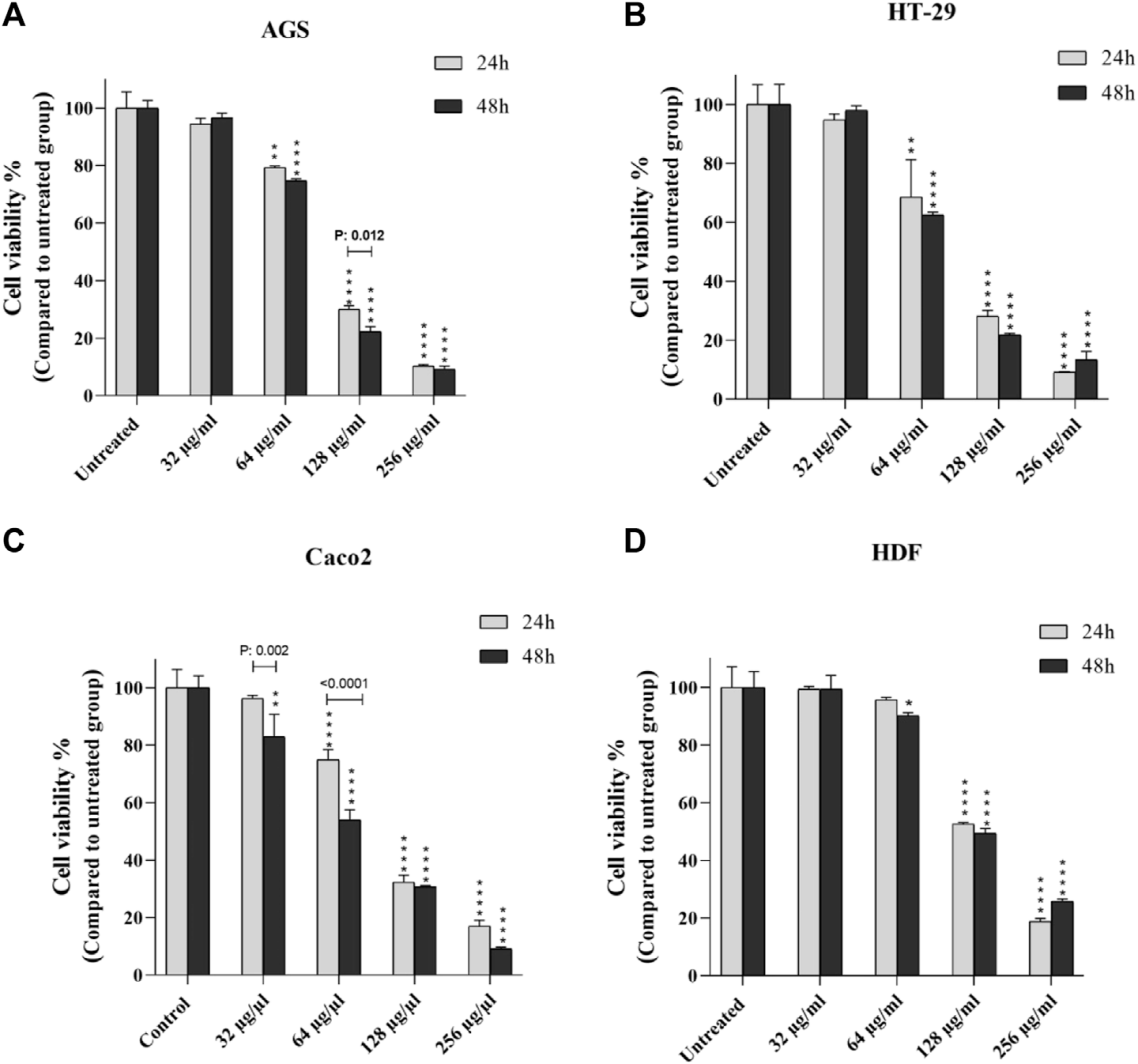
Comparison of cell viability of cancer cells **(A-C)** and normal cells **(D)** after 24 and 48 h exposure to certain concentrations of *T. canis* ES-derived peptide; in Caco 2 cells, the concentration of 32 µg/ml was the starting point for the effect of the peptide on cell viability compared to normal cells, while in the other two cancer cell lines, this starting concentration was 64 µg/ml (*p < 0.05; **p < 0.01; ***p < 0.001; ****p < 0.0001).

Cell viability/mortality and proliferation of peptides were calculated at 24 and 48 h at increasing concentrations in each cell line (Figure 1). There was a direct relationship between the increase in concentration and the increase in mortality, and in all cell lines with a concentration of 64 μg≤, the CV rate was significantly reduced. After 24 h, concentrations of 128 and 256 μg showed the highest effect in all cell lines (*p* < 0.0001). The effect of the peptide on cells appeared to be in a dose-dependent manner. After 48 h of peptide exposure, as in the previous pattern, CV decreased at 64, 128, and 256 μg concentrations in contrast to incremental doses. In Caco-2 cells, the concentration of 32 μg also exhibited significant mortality induction compared to the control group (*p* < 0.01). CV rates were estimated at different concentrations according to the time elapsed for each cell line. As shown in Figure 1, we found that the peptide effect on all cancer cells was observed in a time and dose-dependent manner.

**FIGURE 2:**
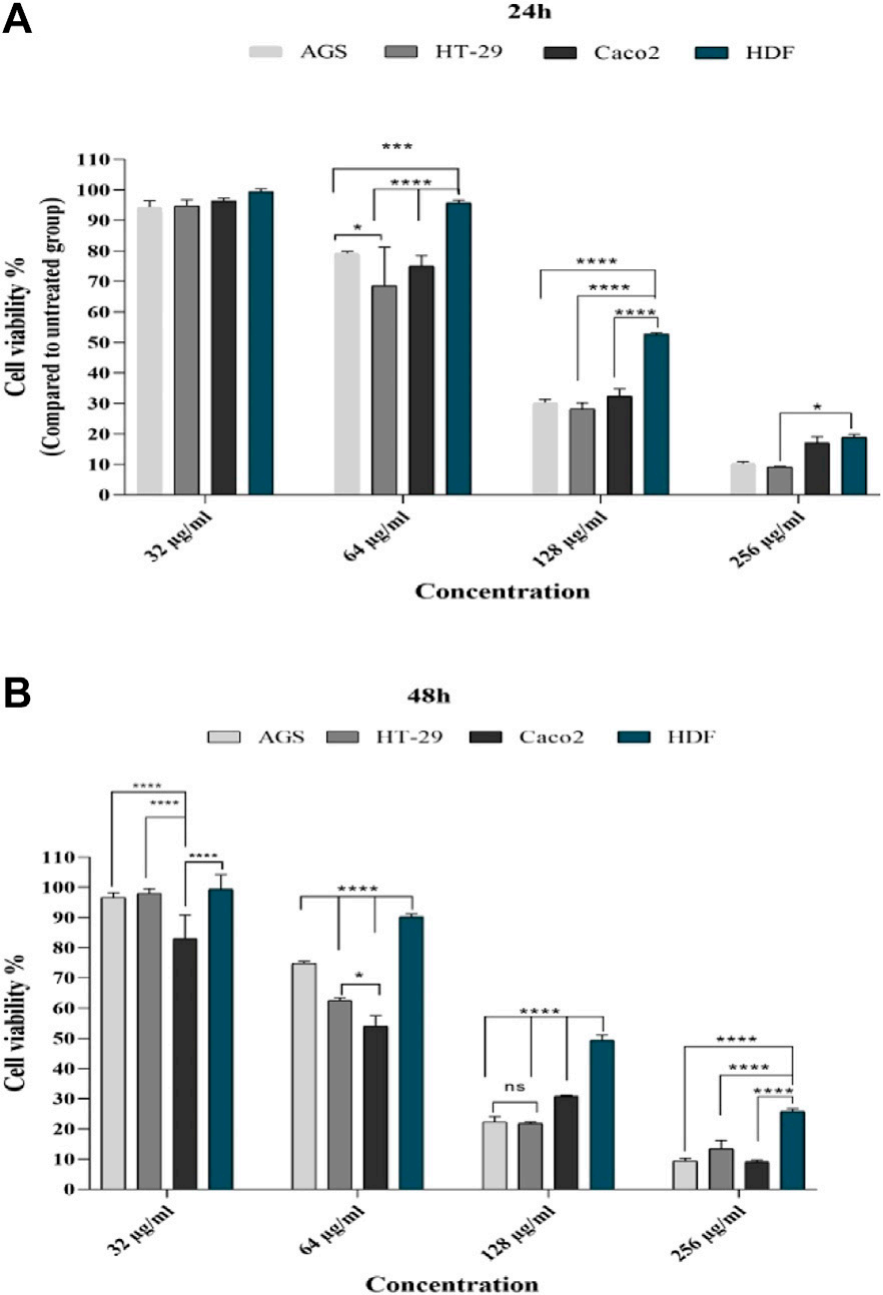
Comparison of cell viability in cell lines with different concentrations of *T. canis* ES TPP after 24 **(A)** and 48 h **(B)** of exposure; after 24 h, at a concentration of 32 μg/ml, cancer cells had a decrease in cv compared to normal cells (not statistically significant); a significant difference was observed from the concentration of 64 μg/ml; after 24 h, the highest effect was in the concentration of 128 μg/ml and the lowest effect was in the concentration of 256, while after 48 h, the effect of 256 μg/ml along with other concentrations was very significant (ns, not significant; *p < 0.05; **p < 0.01; ***p < 0.001; ****p < 0.0001).

At mentioned concentrations, the CV rate in each cell line was compared. At the end of 24 h, the reduction of CV at a concentration of 64 μg in Caco-2, HT-29, and AGS cells was significant compared to the normal cell line (*p* < 0.0001, *p* < 0.01, and *p* < 0.01, respectively); at 128 μg/ml concentration, in all cell lines, CV had a significant reduction compared to the control group (more details are provided in Figure 2).

### 3.2 Half-maximal inhibitory concentration (IC_50_) for *T. canis* excretory-secretory peptide

**FIGURE 3:**
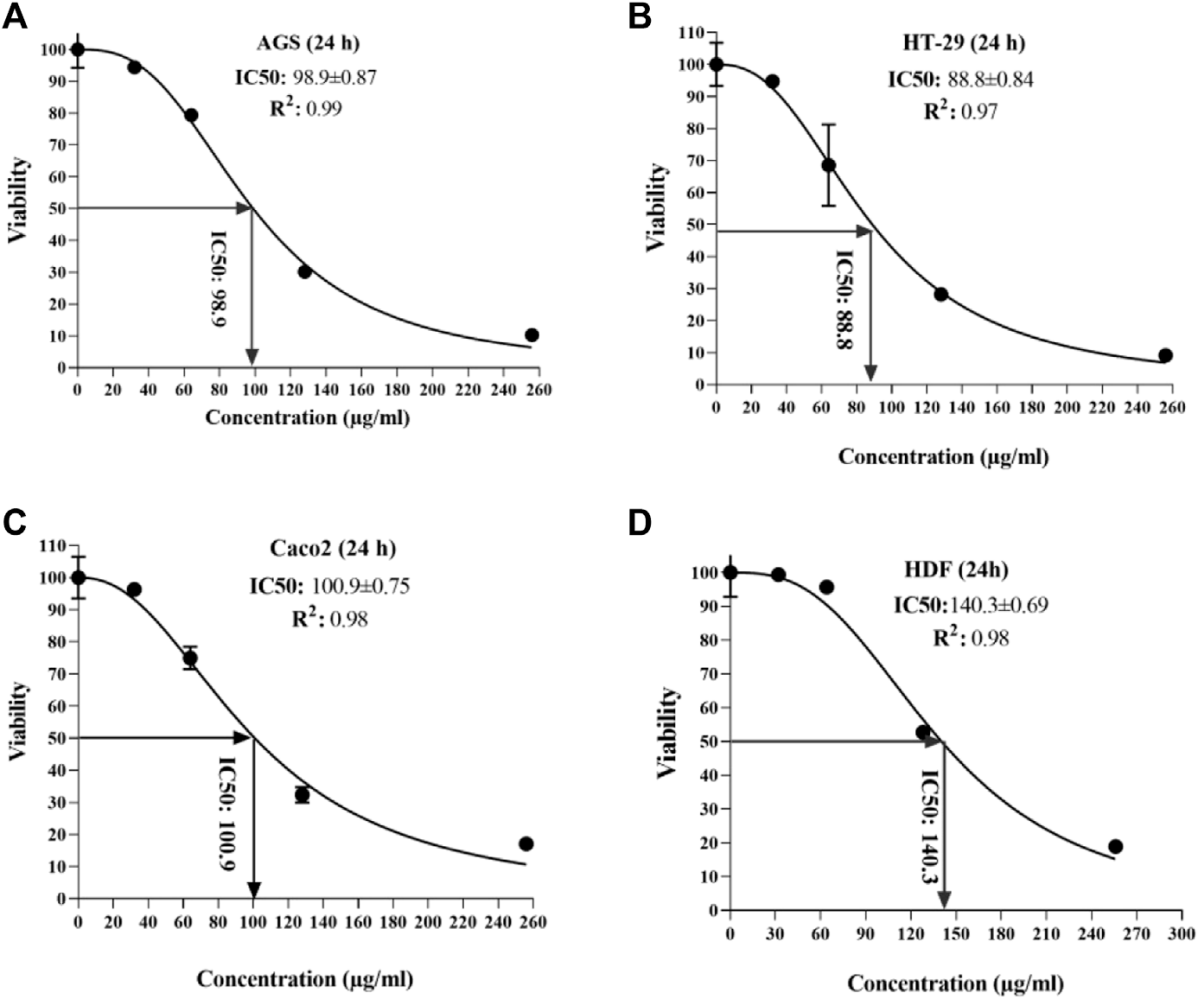
Half-maximal inhibitory concentration of *T. canis* ES TPP in cell lines after 24 h; the highest value was observed in normal cells (140.3) **(D)** and the lowest in HT-29 cells (88.8) **(B)**.

**FIGURE 4:**
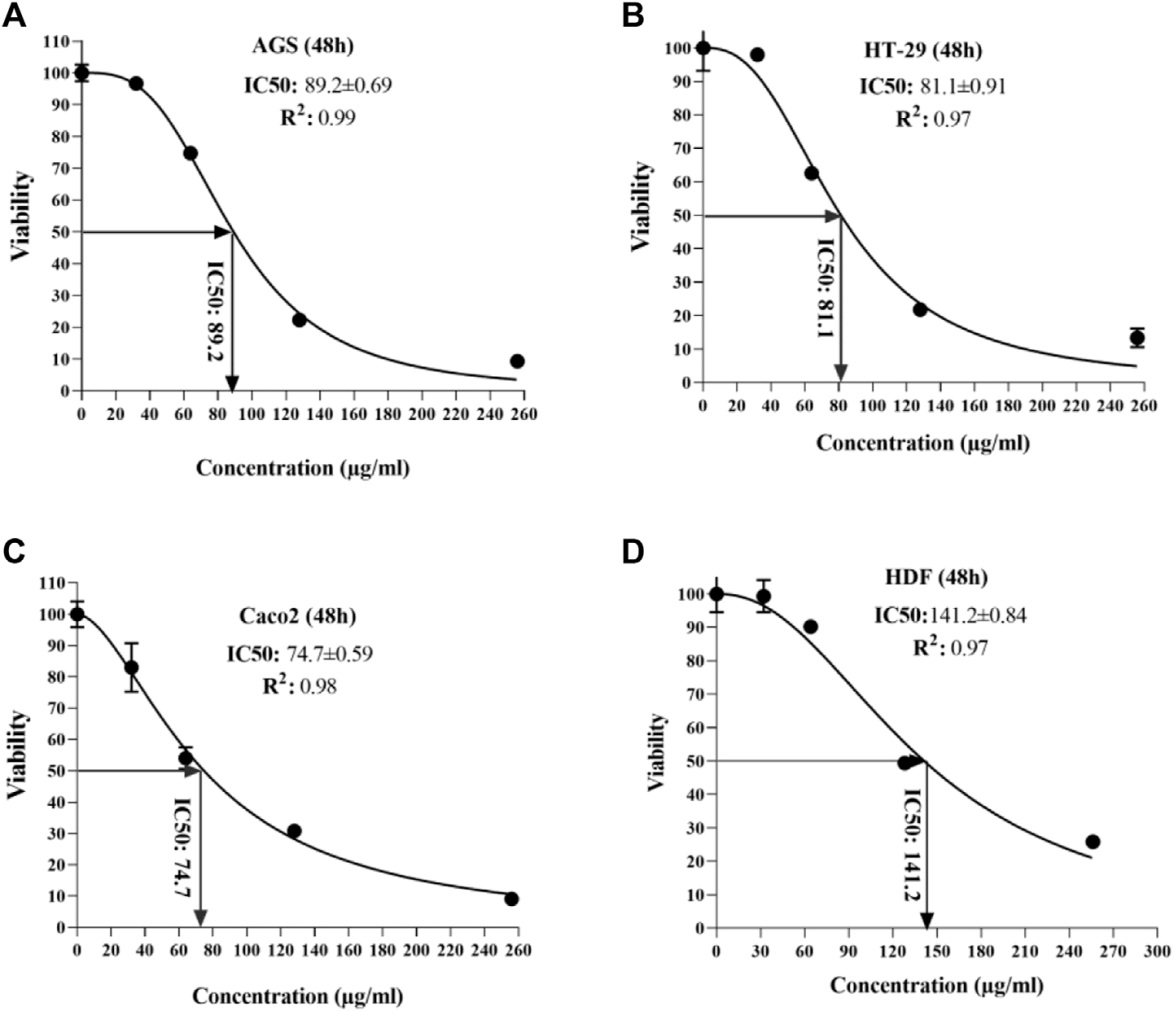
Half-maximal inhibitory concentration of *T. canis* ES TPP in cell lines after 48 h; the highest value was detected in normal cells (141.2) and the lowest in Caco 2 cells (74.7).

According to the observed data from the sample ODs in the MTT assay, IC_50_ was calculated for each cell line at 24 and 48 h after peptide exposure. At the end of the first 24 h, the lowest and highest IC_50_ was seen in the HT 29 cell line (88.8 µg/ml) and HDF cell lines (140.3 µg/ml) (Figure 3); additionally, in the second 24 h, the lowest and highest IC_50_ concentrations were for Caco-2 and AGS (74.7 vs. 89.2 µg/ml, respectively) (Figure 4).

### 3.3 Apoptosis-related factors’ (BCL2, APAF1, and caspase-3) expression levels

In three cancer cell lines, mRNA levels of Bcl2, APAF1, and caspase-3 were evaluated at ascending concentrations and around IC50 concentrations by real-time-qPCR reported by fold change.

**FIGURE 5:**
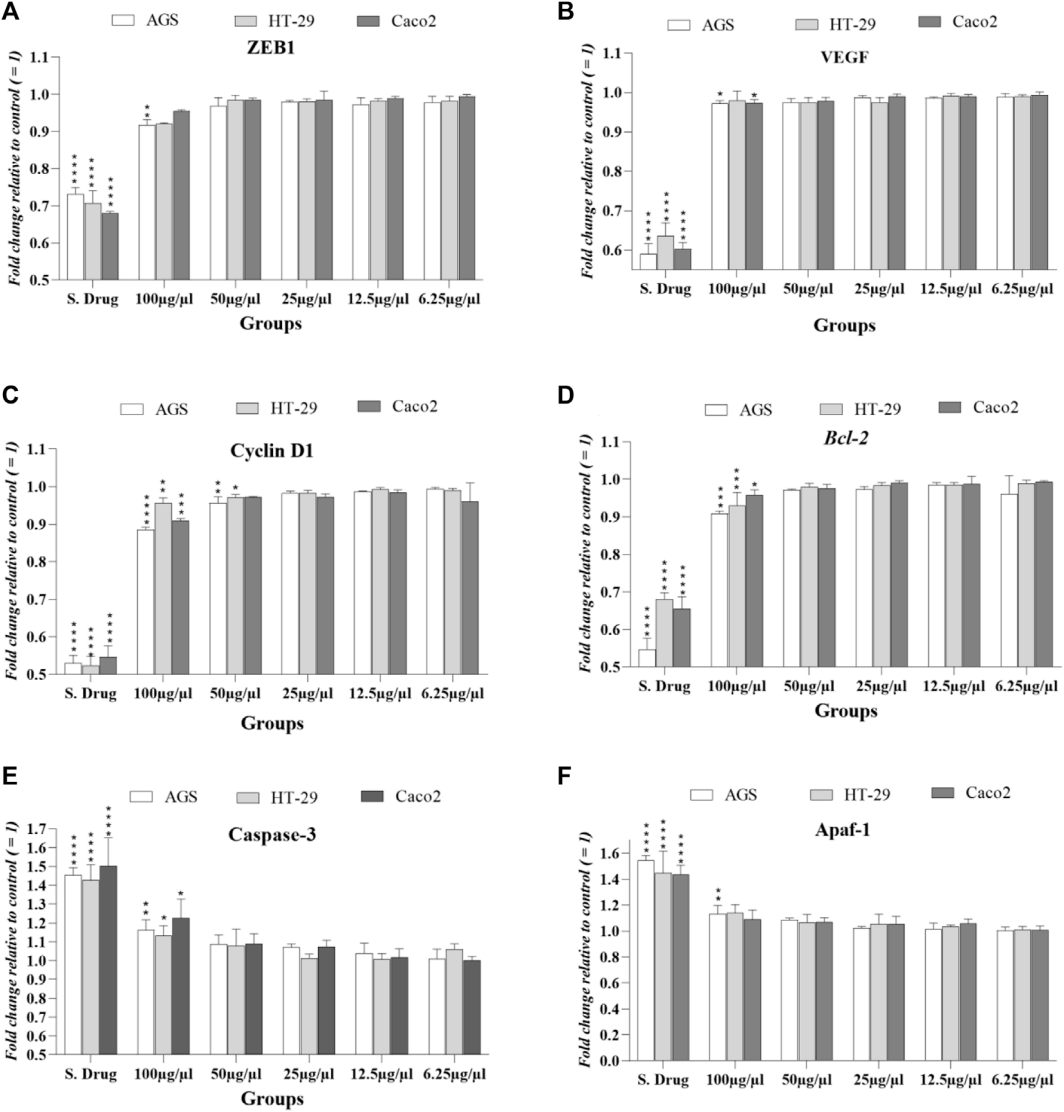
Expression levels of studied factors in three cancer cell lines exposed to *T. canis* ES PP and standard drug (regorafenib) in terms of fold change after 24 h exposure. Major expression changes occurred at 100 μg/ml concentration, and in cyclin D **(C),** 50 μg/ml concentration also induced significant changes in HT-29 and AGS cells; the groups were compared to the untreated group.

As shown in Figure 5, BCL2 expression, which is reduced in cell death (apoptosis), was significantly reduced in the standard drug (regorafenib)-treated group, and only at a concentration of 100 μg/ml (near IC50), has also decreased in all three cell lines. Expression levels of APAF-1, an apoptosis-inducing gene, increased in the standard drug-treated group but showed a significant increase only in the AGS cell line at 100 μg/ml concentration. Regarding the caspase-3 expression in cancer cell lines, it was observed that only at close to IC_50_ concentration (100 μg/ml), a significant increase was observed as caspase-3 is a gene that induces the apoptosis pathway.

### 3.4 Expression levels of cell cycle, metastasis, and angiogenesis regulators (cyclin D1, ZEB1, and VEGF)

The *cyclin D1* gene encodes the cyclin D1 protein that increases in malignancies; in all 3 cell lines treated with *T. canis* peptide, a significant decrease in cyclin D1 expression was observed at a concentration of 100 μg/ml. The peptide at 50 μg/ml also reduced the expression of this gene in HT-29 and AGS cells (*p* < 0.05 vs. *p* < 0.01). The EMT-activator ZEB1 level decrease was seen only in AGS cells (conc: 100 μg/ml; *p* < 0.01).

This significant reduction in the angiogenesis-related gene (VEGF) occurred only in the Caco 2 and AGS cell lines at the highest concentration (100 μg/ml; *p* < 0.05) (details can be seen graphically in Figure 5).

## 4 Discussion

Nowadays, the studies of alternative cancer therapies with natural substances instead of chemical drugs still continue. In addition to plant compounds, the potential of various animal products against the growth of cancer cells is also evaluated (Lin et al., 2020). Parasites, as one of the oldest living organism alongside humans, have long had a close relationship with humans. In the present study, compounds secreted by the *Toxocara* parasite were measured against induction of apoptosis and inhibition of gastrointestinal cancer cell line growth. The results compared to the standard drug. Although at low concentrations, it was not lethal, at high concentrations it showed promising effects on cancer cell mortality and changes in expression levels of the studied factors. Also, the selected *T. canis* ES TPP was able to induce a higher mortality rate than the untreated group, which was in line with the findings of Daneshpour et al. (2019); according to this study, hydatid cyst wall antigens were able to induce apoptosis and inhibition of melanoma cancer growth and proliferation in an animal model.

Protein profiles of *T. canis* secretory–excretory antigens have been identified that contain a diverse range of proteins; the use of crude antigens, despite their ease of use, complicates the finding inference due to the presence of a wide range of proteins. However, in previous studies, Darani et al. (2009) claimed that the crude antigens of *T. canis* eggs showed antitumor potential. The anti-cancer potential of parasitic products is mostly investigated in crude form; a study by Batmonkh et al. (2006) looked at the antitumor properties of parasites (*Trypanosoma cruzi*) in a mouse model in which parasite lysates were able to reduce the tumor size compared to the control group. The present study focused on a specific part of *T. canis* ES proteins that do not interpret the results as misleading.

As mentioned, in the present study, despite the predictions in the online environment, a slightly unexpected, high concentration of peptide is required to kill the cancer cells and induce cell death. However, we believe that compounds with anti-cancer potential can be optimized, including the use of nanoparticles, the use of adjuvants, or combinations with materials that have better delivery to the target cells/tissue (Lv et al., 2021). Choosing the most similar part to anti-cancer ingredients instead of crude proteins seems reasonable. Currently, the use of a computer database can help predict the anti-cancer potential of peptides (Manavalan et al., 2017), as used in the present study (unpublished data). In addition to determining the similarity to previous anticancer compounds, it is also important to determine if the peptide is non-allergic (Vijayakumar and Ptv, 2015; Wei et al., 2018).

Evaluation of cell death in cancer cells is possible by colorimetric methods (MTT assay) and at the molecular level, which in the former is less sensitive and specific, but in the latter, includes the measurement of metastatic, angiogenic, and apoptotic factors such as Bcl-2, APAF1, ZEB1, VEGF, cyclin-D1, and caspase-3 by real-time PCR method, the more accurate test. Bcl-2 (B-cell lymphoma 2) has long been recognized for its role in programmed cell death (apoptosis); it is specifically an inhibitor of apoptosis (Yip and Reed, 2008), and its expression is high in living cells. As the previous gene, APAF1 is a vital factor involved in the internal (mitochondrial) pathway of the apoptosis process, unlike the previous one; in apoptosis, we see an increase in its expression (Cain et al., 2002). *ZEB1* is a multifunction and vital gene encoding the zinc finger E-box binding homeobox 1 protein, involved in many embryonic development processes and tumor progression/metastasis through epithelial–mesenchymal transition (EMT). ZEB1 is necessary for the organ’s formation/development in the embryonic period. Evidence suggests that abnormal expression of ZEB1 may be involved in gastrointestinal (colorectal) cancer (Zhang et al., 2013; Zhang et al., 2015).

In this regard, VEGF is a homodimeric glycoprotein (≈45 kDa M.W). It is the angiogenesis main mediator, which stimulates angiogenesis in embryonic development and is imperative in adult wound healing. Also, VEGF is the vital mediator of cancer angiogenesis, in which it is upregulated by oncogene expression (Ferrara, 2005). Nowadays, cyclin D1 has also been well characterized as a component of DNA repair machinery in human cancer cells (Zhang et al., 2012); significant changes in the expression of this factor due to natural substances (e.g., parasitic products) other than commercial drugs, can promise an innovative therapeutic option. In the two concentrations, we observed a significant difference in expression in cancer cell lines in the present study.

Caspase-3 is an apoptotic and non-apoptotic mediator that is often used as a marker for the efficacy of cancer therapy (Zhou et al., 2018). The peptide used in our study was also able to change the expression levels of this gene at a concentration of 100 μg/ml.

Several mechanisms are hypothesized for the effect of ACPs on tumor cells. The peptide used in the present study, probably in addition to inducing apoptosis in cancer cells, can exert its effect through other mechanisms such as damage to the cell membrane structure, which needs more studies to clarify the details. It was already mentioned that according to the computer-based tools and/or bioinformatical algorithm predictions in the anticancer peptide database (CancerPPD), this peptide has 93% similarity and homology to previously used anti-cancer substances.

Considering the evidence and findings of previous studies, according to the Darani et al. study, the hypothesis that chronic parasitic infections with low parasitic load can lead to immunological changes to induce anti-cancer activities in favor of the host is further strengthened (Darani and Yousefi, 2012).

Due to the fact that *T. canis* eggs are hatched in the intestine, most of the secretions and excretions of larvae occur in the intestine and viscera (liver, lungs, etc.); consequently, studying the malignancies of these organs can be more relevant. Based on the “hygiene hypothesis” and “old friend” hypothesis and combining the findings of previous studies on chronic infections, it may be argued that parasites with a very long biological history can be indeed a hidden source with anti-cancer potential (Oikonomopoulou et al., 2013; Garn et al., 2021). Of course, this claim must be verified, and their use must be safe and optimized.

The present study faced limitations including the inability to synthesize and purify peptides in the country, and there was also limited funding for more specific tests, such as measuring the entry of peptides into cells (by electron microscopy) and measuring the expression of proteins by blotting tests.

## 5 Conclusion

The findings indicated that *T. canis* peptide, at high concentrations, had an effect on cancer cell mortality and altered apoptosis, metastasis, and angiogenesis-related gene expression. However, despite the outcomes of computer-based studies, low concentrations had no significant effect on cancer cell mortality. However, future studies can be promising if the peptide is optimized.

As a suggestion for future studies, the peptide should be combined with nanomaterials or compounds that have anti-cancer properties synergistically. We also suggest performing tests to test in animal models that are experimentally tumorigenic. Moreover, measurement of apoptotic and anti-apoptotic proteins family by the Western blotting technique seems to be helpful.

## Data Availability

The original contributions presented in the study are included in the article/Supplementary Material; further inquiries can be directed to the corresponding author.
